# A Case of Colic Followed by Enteritis Ulceration of Stomach and Possible Perforation

**Published:** 1882-07

**Authors:** 


### VII.—A CASE OF COLIC FOLLOWED BY ENTERITIS ULCERATION OF STOMACH AND POSSIBLE PERFORATION.

REPORTED BY DRS. WALTON AND M'LELLAN

A horse, about twelve years old, was brought to the hospital showing symptoms of colic. The usual remedies were administered, with negative results. Further inquiry into the previous history elicited the fact that the horse had been fed apples at the last feeding. Further examination revealed acidity of the stomach and eructations of fœtid gases. Sodi bicarbonates and cathartics were administered. The pain soon became continuous, which was partially removed by hypodermic injections of morphine. At the end of fourteen hours the severer symptoms had nearly disappeared, he appeared weak and refused food.

An attempt was again made to move the bowels. Oleum lini was freely administered, and later magnesia sulphatis, but without the desired result. The animal continued in this condition for about four days. At the end of this time min. x. of oleum tiglii in oleum lini was tried. This opened the bowels, and free movements followed. The horse now commenced to eat a little soft food, but continued dull in spirits for two days after.

On the morning of the seventh day from the onset of the attack he developed marked symptoms of violent pain, and died in less than two hours.

Necropsy shortly after death. The only marked lesions were a pronounced congestion of the whole alimentary canal, deep and extensive ulceration of the mucous membrane of the stomach.

COLUMBIA VETERINARY COLLEGE HOSPITAL, NEW YORK CITY, 1882.

[ The cause of death in this case seems a little doubtful, but we think it can be explained by the extreme prostration of the nervous system, or one of the ulcers in the stomach may have perforated the peritoneal cavity allowing the escape of gases and not of solid matter, which in itself would be a sufficient cause of death.—ED.]

